# Performance Evaluation of a Solar-Assisted Multistage Heat Pump Drying System Based on the Optimal Drying Conditions for *Solanum lycopersicum* L.

**DOI:** 10.3390/foods14071195

**Published:** 2025-03-28

**Authors:** Yimin Tang, Xiaoqiong Li, Peng Xu, Junling Yang, Zhentao Zhang, Ruixiang Wang, Dandan Zhao, Ramadan Elgamal

**Affiliations:** 1 Beijing Engineering Research Centre of Sustainable Energy and Buildings, School of Environment and Energy Engineering, Beijing University of Civil Engineering and Architecture, Beijing 100044, China; 18640468327@163.com; 2 Technical Institute of Physics and Chemistry, Chinese Academy of Sciences, Beijing 100190, China; lixiaoqiong@mail.ipc.ac.cn (X.L.); yangjl@mail.ipc.ac.cn (J.Y.); zzt@mail.ipc.ac.cn (Z.Z.); 3 Key Laboratory of Equipment and Energy-Saving Technology on Food & Pharmaceutical Quality Processing, Storage and Transportation, China National Light Industry, Beijing 100190, China; 4 College of Food Science & Biology, Hebei University of Science & Technology, Shijiazhuang 050018, China; zdd2016@hebust.edu.cn; 5 Agricultural Engineering Department, Faculty of Agriculture, Suez Canal University, Ismailia 41511, Egypt; ramadan_emara@agr.suez.edu.eg

**Keywords:** *Solanum lycopersicum* L., solar assisted heat pump drying, drying characteristics, drying quality, system efficacy

## Abstract

This study aims to evaluate the drying performance of a multi-stage solar-assisted heat pump drying system for tomatoes. The method involves theoretical calculations based on the optimal drying process and experimental investigations to assess the impact of different drying temperatures and relative humidity on drying characteristics. The results from the theoretical calculations reveal that the multi-stage solar-assisted heat pump drying system outperforms a single-stage system, particularly under lower ambient temperatures or higher fresh air volumes. In spring/autumn, with 25% fresh air, solar energy accounts for 85.12% of the total energy consumption, achieving a performance coefficient of 39.16, a moisture extraction rate of 40.7 kg/kWh, and energy consumption of 0.02 kWh/kg. Carbon dioxide emissions amount to 10.45 kg/year, with a net reduction of 7.88 kg/year. The experimental results indicate that higher relative humidity increases drying time and reduces the diffusion coefficient, which results in higher material temperatures and greater nutrient loss. The optimal drying process is achieved at 70 °C and 20% relative humidity. In conclusion, the multi-stage solar-assisted heat pump drying system demonstrates superior performance in energy efficiency and sustainability compared to single-stage systems. The optimal drying conditions for tomatoes are identified, and the findings contribute to improving drying processes in food preservation while minimizing environmental impact.

## 1. Introduction

*Solanum lycopersicum* L., commonly known as tomato, is widely cultivated and consumed fresh or processed into various food products [1]. Global annual tomato production exceeds 400,000 tons, but fresh tomatoes are highly perishable and seasonal variations limit year-round availability, making drying a critical aspect of tomato slice processing [2]. The global demand for dried tomatoes and other processed tomato products is steadily increasing, driven by the growing need for long shelf life, food security, and the rise in popularity of convenience foods. The dried tomato market alone is projected to grow by 6.5% annually, reaching a value of over USD 1.5 billion by 2027. The aim of drying is to remove moisture from products to extend shelf life, reduce weight, and improve quality and economic value. Over 85% of industrial thermal dryers in the food industry are conventional, accounting for 12~20% of total energy consumption, but with only 30% energy efficiency, while contributing to 90% of total processing costs [3]. Approximately 35~45% of the energy is lost as hot exhaust gases, resulting in high energy wastage and significant greenhouse gas emissions. In the context of the “carbon peaking and carbon neutrality” goals, the food industry is not only focusing on improving existing drying technologies, but is also actively developing alternative methods to achieve energy savings and emissions reductions, thereby promoting sustainable development [4]. Addressing the environmental and economic challenges in tomato drying processes is crucial for ensuring global food security, improving nutritional outcomes, and aligning with market trends towards more sustainable and energy-efficient food production methods. The reduction in carbon dioxide emissions in drying processes is of paramount importance in addressing climate change and meeting global sustainability targets. By optimizing energy use and minimizing waste, these systems can significantly contribute to carbon neutrality.

It is important to note that drying technologies are highly dependent on environmental factors such as ambient temperature and humidity. These factors can affect drying efficiency and the quality of the dried products. The variability of environmental conditions also introduces challenges in achieving consistent results, particularly under fluctuating sunlight and seasonal changes.

Solar energy, as a renewable resource, received increasing attention for its applications in various technologies, including drying processes. Solar drying (SD) technology uses solar radiation as a heat source to evaporate moisture from materials by air convection to achieve the drying effect, which is applied in the processing of agricultural products [5]. Different solar drying technologies, such as direct solar drying, indirect solar drying, and mixed-mode solar drying, offer varying levels of energy efficiency and drying performance. Indirect and mixed-mode systems typically provide better energy efficiency as they control temperature and humidity more effectively, preventing overheating and quality loss. Direct solar dryers, though simpler and cost-effective, are less energy-efficient, particularly under variable sunlight conditions. Djebli studied the process of drying potatoes using an indirect and a mixed solar dryer and a comparison of their respective performances. Experimental results show that the mixed solar dryer had the slowest drying rate compared to the indirect solar dryer with drying times of 4.75 h and 3.67 h, respectively [6]. Energy consumption in solar dryers depends on system design. Indirect systems tend to be more efficient due to minimized heat loss and the ability to operate under low sunlight. In contrast, direct solar dryers are more prone to energy inefficiency during fluctuating solar radiation or excessive exposure. Fudholi developed an SD system consisting of a vacuum tube solar collector and a drying chamber with a specific energy consumption of 5.26 kWh/kg, an efficiency of 13%, and an average thermal efficiency of 57% [7]. Recent advancements in solar drying include integrating passive and active systems. These systems optimize energy use by recovering residual heat, especially in variable environmental conditions, and feature advanced control systems for better temperature, humidity, and airflow management, ensuring efficient and uniform drying.

However, solar drying systems often face repeatability issues, as performance can vary based on daily and seasonal fluctuations. Additionally, the quality of dried products can degrade if environmental factors such as sunlight and humidity are inconsistent, leading to poor uniformity in drying. Furthermore, nighttime humidity reabsorption is a common issue, where moisture from the surrounding environment can be reabsorbed by dried materials, potentially reducing shelf life and quality.

Research shows that SD has several advantages, including energy efficiency, low operating costs, and simple equipment. However, SD systems have the problem of venting wet exhaust gas directly to the atmosphere, resulting in energy wastage. In addition, SD cannot precisely control the temperature and humidity of the hot drying air, resulting in poor-quality dried products. To overcome these problems, researchers proposed and investigated heat pump-assisted SD systems.

A heat pump is a device that uses the principle of heat transfer to move thermal energy from a low-temperature environment to a high-temperature environment. The advantage of a heat pump is that it allows precise control of temperature and humidity during the drying process and can efficiently recover residual heat after drying, resulting in energy savings and environmental benefits [8]. Kuan established a numerical model to predict the energy performance of a heat pump-assisted solar dryer applied to banana drying in a continental climate. The model takes into account the synergistic effects of the solar air collector, drying chamber, and heat pump system. Through this model, Kuan found that the specific moisture extraction rate (*SMER*) and coefficient of performance (*COP*) were approximately 0.6 kg/kWh and 2.72, respectively, indicating higher energy efficiency compared to conventional solar dryers [9]. Wang developed a solar-assisted heat pump drying (SAHPD) system with secondary heat recovery, which was applied to mango drying and showed a reduction in power consumption of 3.5 kW/h with a *COP* of 3.69 and an average heat recovery efficiency of 41.7% for the heat exchanger during operation [10].

Moreover, heat pump-assisted systems can help reduce sample contamination risks, as they are typically designed to operate in a more controlled environment. However, care must be taken to prevent contamination through improper handling or exposure to unsanitary conditions.

Studies have shown that incorporating heat pumps into SD systems for drying agricultural products can improve efficiency by utilizing heat recovery, leading to energy savings. However, there are several limitations to existing research. On the one hand, studies on the performance of drying systems do not take into account the conditions required for material drying, which makes it difficult to guarantee the quality of dried materials under high system performance conditions. On the other hand, studies show that although the closed single-stage heat pump system is more effective in recovering waste heat, this system mode leads to low heat pump compression ratio, insufficient waste heat recovery, and unreasonable contribution rate of energy provided by solar energy and heat pump, and the system performance cannot reach the optimum. Additionally, food safety must be carefully considered, as improper drying can lead to microbial growth or contamination. This concern underscores the importance of maintaining a sterile environment and ensuring that drying processes meet regulatory standards to ensure the safety of the final product.

Therefore, in this study, a semi-open multi-stage solar-assisted heat pump drying (SAHPD) system was designed, which represents a novel approach in tomato slice processing. Unlike traditional single-stage systems, the semi-open multi-stage configuration allows for more precise control over temperature and humidity during the drying process. This approach enhances energy efficiency by recovering residual heat at each stage and optimizing the drying environment. By incorporating a heat pump-assisted subsystem, the system can better handle variations in ambient conditions, ensuring consistent drying performance and improved product quality. This method is innovative because it combines the advantages of solar drying with advanced heat pump technology to maximize energy use, minimize waste, and improve the drying uniformity of tomato slices. The development of this system could advance existing drying technologies, pushing towards more sustainable, energy-efficient, and environmentally friendly drying processes in the agricultural sector.

The research aims to enhance the quality of dried agricultural products while reducing energy consumption, contributing to carbon peak and carbon neutrality goals in agricultural processing. The main aspects of the study include the following: (1) Investigating the effects of different drying temperatures (50 °C, 60 °C, and 70 °C) and relative humidities (20%, 40%, and 60%) on drying characteristics, material temperature, effective moisture diffusion coefficient, and activation energy of tomato slices, ultimately identifying the optimal drying process. (2) Evaluating the performance of the multi-stage SAHPD system—including specific energy consumption (*SEC*), specific moisture extraction rate (*SMER*), coefficient of performance (*COP*), energy consumption, and carbon emissions—under varying ambient conditions (summer and spring/autumn) with fresh air volume ratios of 10%, 15%, 20%, and 25%, to determine the optimal operating conditions and system configuration.

## 2. Materials and Methods

### 2.1. Materials and Drying Conditions

Fresh Provence tomatoes were purchased from a market in Hebei, China, and carefully selected to ensure uniform size, with an average diameter of 6.0 ± 0.2 cm and a thickness of 0.5 ± 0.1 cm. The tomatoes exhibited similar ripeness, color, and firmness. All samples were free from mechanical damage, decay, or disease, with smooth surfaces and uniformly red or orange-red coloration. The ripeness level was classified as 8–9, with firm flesh, small seed cavities, and moderate moisture content, ensuring consistency in experimental conditions and repeatability in the drying process. The initial moisture content of fresh tomato slices is determined to be 94% ± 2% according to GB 5009.3-2016 National Food Safety Standard Determination of moisture in foods [11].

The experiment uses a full factorial design, with temperatures chosen as 50 °C, 60 °C, and 70 °C and relative humidity set at 20%, 40%, and 60%, all at an air speed of 1 m/s. A total of nine different drying conditions were studied based on pairwise combinations of these parameters. Samples were taken every 30 min for color and weight measurements. A colorimeter was used for color measurements (CR-400, Konica Minolta, Tokyo, Japan), an electronic balance (Mettler Toledo MS Series, Greifensee, Switzerland) for weight measurements, and temperature was continuously monitored in real time with a probe thermometer (Testo 925, Lenzkirch, Germany).

### 2.2. SAHPD System

**Figure 1:**
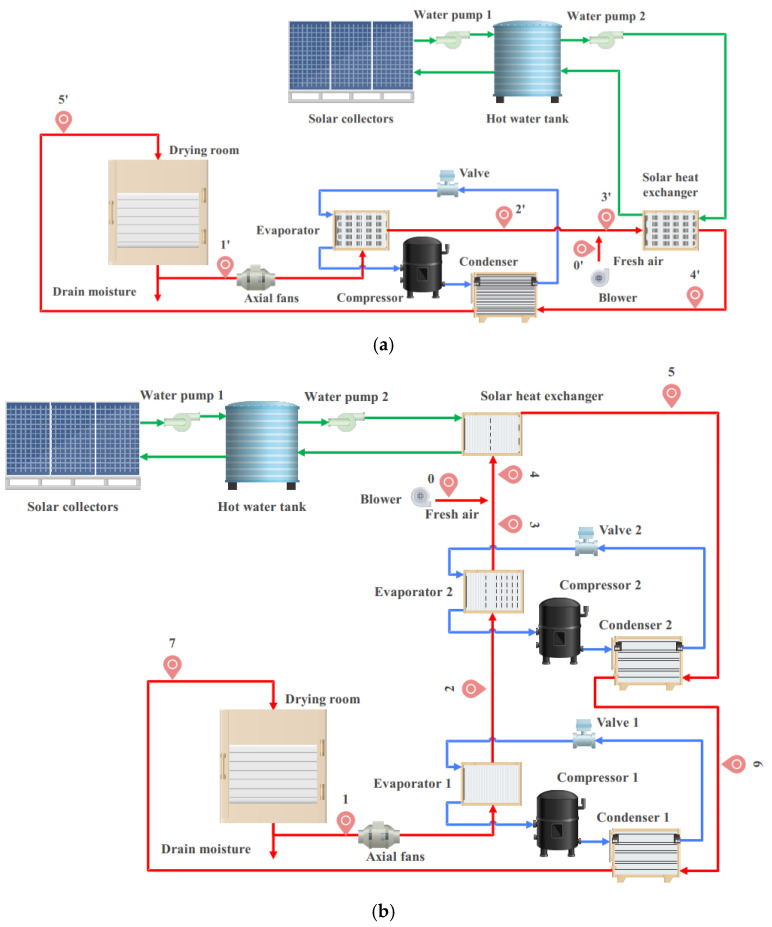
Schematic diagram of the multi-stage SAHPD system: (**a**) single-stage SAHPD system; (**b**) double-stage SAHPD system.

Figure 1 shows the SAHPD system, which consists of a solar drying (SD) subsystem and a heat pump drying (HPD) subsystem. The SD subsystem includes flat-plate solar collectors, a thermal storage tank, a heat exchanger, and control valves, while the HPD subsystem uses R134a refrigerant with components such as an evaporator, condenser, compressor, expansion valve, and fans. This system uses solar energy to dry tomato slices and stores the heat in the thermal tank to ensure a continuous supply. The standard for activating the HPD system is typically triggered when the SD subsystem is unable to meet the heat demand. When the heat provided by the SD subsystem falls below a predefined minimum heat requirement, the HPD subsystem is activated to supply additional heat. The numerical thresholds are determined based on the drying process’s thermal load, the flow of moist air, and the properties of the material being dried. For example, when the heat output of the SD system is less than 50% or the heat source temperature falls below a certain set point, the HPD system automatically starts. The precise selection and sizing of these thresholds ensure that the HPD system activates under optimal conditions to enhance energy efficiency. Moist air from the drying process then passes through the evaporator and condenser for secondary waste heat recovery, improving energy efficiency. The system operation calculations are based on the following conditions: The location is set to Beijing, with a summer temperature of 30 °C and a spring/autumn temperature of 25 °C. The solar radiation intensity is taken as 8000 kJ/m^2^, with the solar duration meeting the drying time requirements. These data provide a basis for optimizing system operation and help more accurately adjust the activation conditions of the HPD system. The specific selection and sizing of the subsequent system is based on the different processing volumes and corresponding loads.

**Figure 2:**
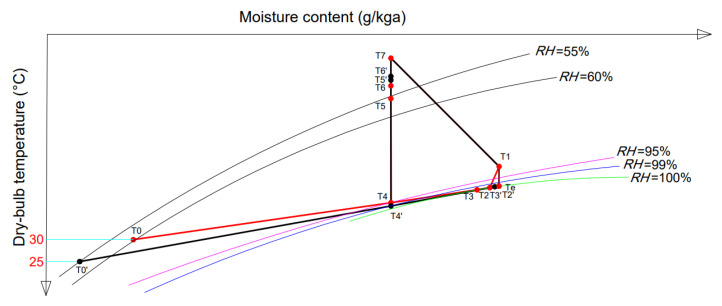
Psychrometric chart of the multi-stage SAHPD system.

Figure 2 shows the psychrometric graphs for the double-stage SAHPD system in summer and spring/autumn. In summer, points *T*_7_ to *T*_1_ show the dehumidification of hot air in the kiln, where the temperature decreases and the humidity increases. Points *T*_1_ to *T*_e_ represent cooling at constant humidity to saturation (*RH* = 100%) and points *T*_e_ to *T*_2_ represent cooling and dehumidification at constant *RH*. Points *T*_2_ to *T*_3_ represent cooling and dehumidification by the second-stage heat pump evaporator. *T*_3_ then mixes with fresh air (*T*_0_) to form *T*_4_. Points *T*_4_ to *T*_5_ and *T*_5_ to *T*_6_ represent the heating processes as the hot air passes through the first- and second-stage heat pump condensers, respectively. Finally, *T*_6_ to *T*_7_ represent the heating as the hot air passes through the heat exchanger of the SD subsystem, completing a cycle. *T*_1′_ to *T*_7_ represent the spring/autumn cycle.

### 2.3. Methods of Quality Analysis

#### 2.3.1. Characterization of the Drying Operation

According to the experimental data on the moisture ratio (*MR*) for tomato drying, the *MR* can be expressed as follows [12]:

(1) $$MR\left(db\right)=MC_{t}/MC_{i}$$

where *MC*_t_ is the moisture content (d.b.) at a given time, kg/kg. *MC*_i_ is the initial moisture content of the material, kg/kg.

The drying rate (*DR*) of the material is calculated as follows:

(2) $$DR=dMC/dt=\left(MC_{t+\mathrm{dt}}-MC_{t}\right)/dt.$$

The water diffusivity of the biological material is generally evaluated using a simplified version of Fick’s second law, known as Fick’s diffusion. Expressed as [13].

(3) $$\partial MR/\partial t=D_{\mathrm{eff}}\nabla^{2}MR$$

(4) $$MR=\frac{8}{\pi ²}exp\left[-\frac{\pi ²D_{\mathrm{eff}}t}{4L^{2}}\right]$$

where *L* is the thickness of the slice, m. *D*_eff_ is the effective moisture diffusivity, m^2^/s.

Temperature and diffusivity are related by the Arrhenius equation and are used to estimate the activation energy (*E*_a_).

(5) $$D_{\mathrm{eff}}=D_{o}exp\left(\frac{-E_{a}}{R_{g}\left(T+273.15\right)}\right)$$

where *D*_o_ is the pre-exponential factor. *D*_eff_ is the effective diffusion coefficient, m^2^/s. *R*_g_ is the universal gas constant, with a value of 8.314 J/(mol K). *T* is the drying temperature, K.

#### 2.3.2. Color Quality

Color measurements were made using a color meter (CR-400, Konica Minolta, Tokyo, Japan). Before color measurement, the colorimeter was calibrated using a standard white ceramic plate (L* = 96, a* = 0.13, and b* = 1.63). L* denotes the lightness (L* = 0 for black, L* = 100 for white), a* denotes the intensity in red-green (a* > 0 for red, a* < 0 for green), and b* denotes the intensity in blue to yellow (b* > 0 for yellow, b* < 0 for blue) [14]. Color difference measurements were conducted under incandescent lighting, with the observer using a 10° viewing angle to measure the tomato pulp.

The degree of color change between the fresh and dried tomato paste is indicated by the total color change (∆E). It was calculated according to the following equation [15]:

(6) $$\Delta E=\sqrt{\Delta L^{*2}+\Delta a^{*2}+\Delta b^{*2}}.$$

The browning index (*BI*) is an assessment of the degree of browning caused by enzymatic or non-enzymatic reactions. The calculation of the *BI* is usually based on the chromaticity parameters *L*, *a*, and *b* as measured by the colorimeter [16].

(7) $$x=\left(a+1.75L\right)/\left(5.645L+a-3.012b\right)$$

(8) $$BI=100\left(x-0.31\right)/0.17$$

#### 2.3.3. Phenolic and Flavonoid

The instruments required to measure the total phenol and total flavonoid content in dried tomato slices include a UV-Visible Spectrophotometer (Shimadzu UV-1800, Kyoto, Japan), a Desktop Centrifuge (Eppendorf 5804, Berzdorf, Germany), an Ultrasonic Homogenizer (Qsonica Q700, Newtown, CT, USA), a Water Bath (IKA HB 10 Basic, Staufen, Germany), a Balance (Mettler Toledo MS Series, Switzerland), and Pipettes (Thermo Fisher Scientific Finnpipette F1, Waltham, MA, USA).

To determine the total phenolic content in dried tomato slices, first, the tomatoes are dried to constant weight by drying experiments, crushed, and passed through a 30–50 mesh sieve. About 0.1 g of the sample is weighed and 2.5 mL of ethanol extraction solvent is added. The mixture is extracted using ultrasonic extraction at 300 W power and 60 °C for 30 min. After extraction, the mixture is centrifuged at 12,000 rpm and 25 °C for 10 min. The supernatant is collected and diluted to 2.5 mL with the extraction solvent for further analysis.

Measurement steps: (1) Preheat the spectrophotometer for more than 30 min, set the wavelength to 760 nm, and calibrate with distilled water. (2) Dilute the standard solution of gallic acid (5 mg/mL) with distilled water to concentrations of 0.15625, 0.078125, 0.039, 0.02, 0.01, 0.005, and 0.0025 mg/mL for measurement. (3) Follow the steps in Table 1 for the operation [17].

Vortex and let sit at room temperature for exactly 10 min, then measure the absorbance at 760 nm, recorded as control tube, measurement tube, standard tube, and blank tube. Calculate Δ*A* measurement as the difference between the measurement tube and control tube, and Δ*A* standard as the difference between the standard tube and blank tube. Each measurement tube requires one control tube. The standard curve and blank tube measurements only need to be performed 1–2 times. Establish a standard curve based on the concentration and absorbance Δ*A* standard of the standard tube. Using the standard curve, substitute the Δ*A* measurement into the formula to calculate the sample concentration. The total phenolic content is equal to the sample concentration multiplied by the volume of the extraction solvent and then divided by the sample mass.

To determine the total flavonoid content in dried tomato slices, first, dry the tomatoes to constant weight by drying experiments, grind them, and pass through a 30–50 mesh sieve. Weigh approximately 0.1 g of the sample, add 1 mL of ethanol extraction solvent, and perform ultrasonic extraction with a power of 300 W, 5 s of ultrasonic bursts, followed by 8 s of pause, at 60 °C for 30 min. Centrifuge at 12,000 rpm and 25 °C for 10 min, collect the supernatant, and dilute with extraction solvent to a final volume of 1 mL for analysis.

Measurement steps: (1) Preheat the spectrophotometer for at least 30 min, set the wavelength to 470 nm, and calibrate to zero with distilled water. (2) Preparation of standard solution: dilute the 10 mg/mL rutin standard solution with the standard dilution solution to concentrations of 1.5, 1.25, 0.625, 0.3125, 0.15625, 0.078, 0.039, and 0.02 mg/mL for use. (3) Follow the steps outlined in Table 2 for the procedure.

Mix thoroughly, incubate in a 37 °C water bath for 45 min, then centrifuge at 10,000× *g* for 10 min and collect the supernatant. Measure the absorbance at 470 nm and calculate Δ*A* as the difference between the sample and the control, and Δ*A′* as the difference between the standard and the blank. Plot the standard curve with rutin concentration on the x-axis and Δ*A′* on the y-axis. Use the equation to calculate the sample concentration by substituting Δ*A* into the formula. The flavonoid content is calculated as the sample concentration multiplied by the volume of the extraction solvent and divided by the sample mass.

### 2.4. Methods of Performance Analysis

Based on a system with a tomato slice processing capacity of 80 kg/h and a dehumidification rate of 111 kg, and using the optimal drying process, the ambient conditions in summer are 30 °C and 60% relative humidity, while in spring/autumn, the ambient temperature is 25 °C with a humidity of 55%. Theoretical calculations are conducted for the system, which uses a vertical air supply with a circulating air volume of 7210 kg/h and a total heat load of 71.66 kW, ignoring the power of the pumps and fans. The aim is to perform energy consumption comparison calculations under different drying conditions, providing theoretical guidance for the selection of drying materials and equipment.

#### 2.4.1. Solar Thermal

Calculation of collector efficiency [18].

(9) $$\eta =C_{p}m\Delta t/A_{c}I_{c}=C_{p}\rho VA_{1}\left(t_{o}-t_{i}\right)/A_{c}I_{c}$$

where *C*_p_ is the specific heat capacity of air, J/(kg·K). *m* is the mass flow rate, kg/s. *A*_c_ is the collector area, m^2^. *I*_c_ is the solar radiation intensity or incident energy per unit area, W/m^2^. *ρ* is the density of air, kg/m^3^. *V* is the volume flow rate, m^3^/s. *t*_o_ is the outlet air temperature, °C. *t*_i_ is the inlet air temperature, °C.

The active area of the collector is as follows [19]:

(10) $$A_{e}=\frac{Q}{\bar{HR_{h}}\tau \alpha \eta }$$

where *Q* is the amount of heat required to be supplied by the collector, $\bar{HR_{h}}$ is the intensity of the solar radiation on the inclined plane, *τ* is the transmittance of the collector cover glass, *α* is the absorption rate of the collector absorber plate, and *η* is the instantaneous thermal efficiency value of the collector.

#### 2.4.2. Hot Water Storage Tank

Based on the enthalpy method, the theoretical heat release as follows [20]:

(11) $$Q_{w}=\rho_{w}Vc_{w}\left(T_{s}-T_{b}\right)$$

where *V* is the volume of the hot water storage tank. *ρ*_w_ is the density of water, g/cm^3^. *c*_w_ is the specific heat capacity of water, kJ/(kg·°C). *T*_s_, *T*_b_ are the beginning and end temperatures of the hot water storage tank, °C

The hot water flow is as follows [19]:

(12) $$V_{a}=\Psi \frac{Q}{\rho c_{p}\Delta t}$$

where *V*_a_ is the hot water rate, m^3^/h. *Q* is the collector heat load. *Ψ* is the pump efficiency and water reserve factor. *c*_p_ is the constant pressure specific volume of water, kJ/(kg K). *ρ* is the density of the water, kg/m^3^. ∆*t* is the average collector temperature rise, °C.

#### 2.4.3. Evaluation Indicators of the Overall System

The main reference index for the performance of the drying system is the *COP*. Therefore, *COP*_SAHPD_ reflects the heating performance of the system in the combined drying mode of solar collector and heat pump. The calculation is as follows [21]:

(13) $$COP_{\mathrm{SAHPD}}=P_{\mathrm{cd}}/\left(P_{\mathrm{cr}}+P_{\mathrm{cf}}+P_{\mathrm{cp}}\right)$$

where *P*_cd_ is the heat output of the heat pump condensate, kW. *P*_cr_ is the electrical power of the compressor, kW. *P*_cf_ is the total power of all the circulating fans in the system, and *P*_cp_ is the power of the circulating water pump.

In addition to the *COP* in the formula, *SMER* (kg/kWh) and *SEC* (MJ/kg) are used to evaluate the energy consumption of the drying process [5].

(14) $$SMER=Mw/\left(\int_{0}^{t}P_{\mathrm{cr}}dt+\int_{0}^{t}P_{\mathrm{cf}}dt+\int_{0}^{t}P_{\mathrm{cp}}dt\right)$$

(15) $$SEC=\left(\int_{0}^{t}P_{\mathrm{cr}}dt+\int_{0}^{t}P_{\mathrm{cf}}dt+\int_{0}^{t}P_{\mathrm{cp}}dt\right)/Mw$$

where *M*_w_ is the mass of moisture removed from the material during the drying process, kg. The integrals of *P*_cr_, *P*_cf_, and *P*_cp_ represent the total energy consumption of the system at the end of the drying process, kWh.

The lowest *CO*_2/emission_ is calculated using the following formula [22]:

(16) $$CO_{2/\mathrm{emission}}=0.98E_{m}/n$$

where *E*_m_ is the energy required to produce one kilogram of the material, kWh. *n* is the number of years.

The amount of *CO*_2/mitigation_ of the drying systems (*X*) is defined based on distribution and transmission losses, *L*_d_ = 30% and *L*_t_ = 15%, respectively. Therefore, *X* and *CO*_2/mitigation_ are calculated as follows [23]:

(17) $$X=\frac{1}{1-L_{t}}\times \frac{1}{1-L_{d}}\times 0.98\approx 1.71\mathrm{kg}$$

(18) $$CO_{2/\mathrm{mitigation}}=E_{m}\times X$$

(19) $$NetCO_{2/\mathrm{mitigation}}=CO_{2/\mathrm{mitigation}}-CO_{2/\mathrm{emission}}=\left[E_{\mathrm{out}}\times n\times X-E_{m}\right].$$

#### 2.4.4. Computational Logic

**Figure 3:**
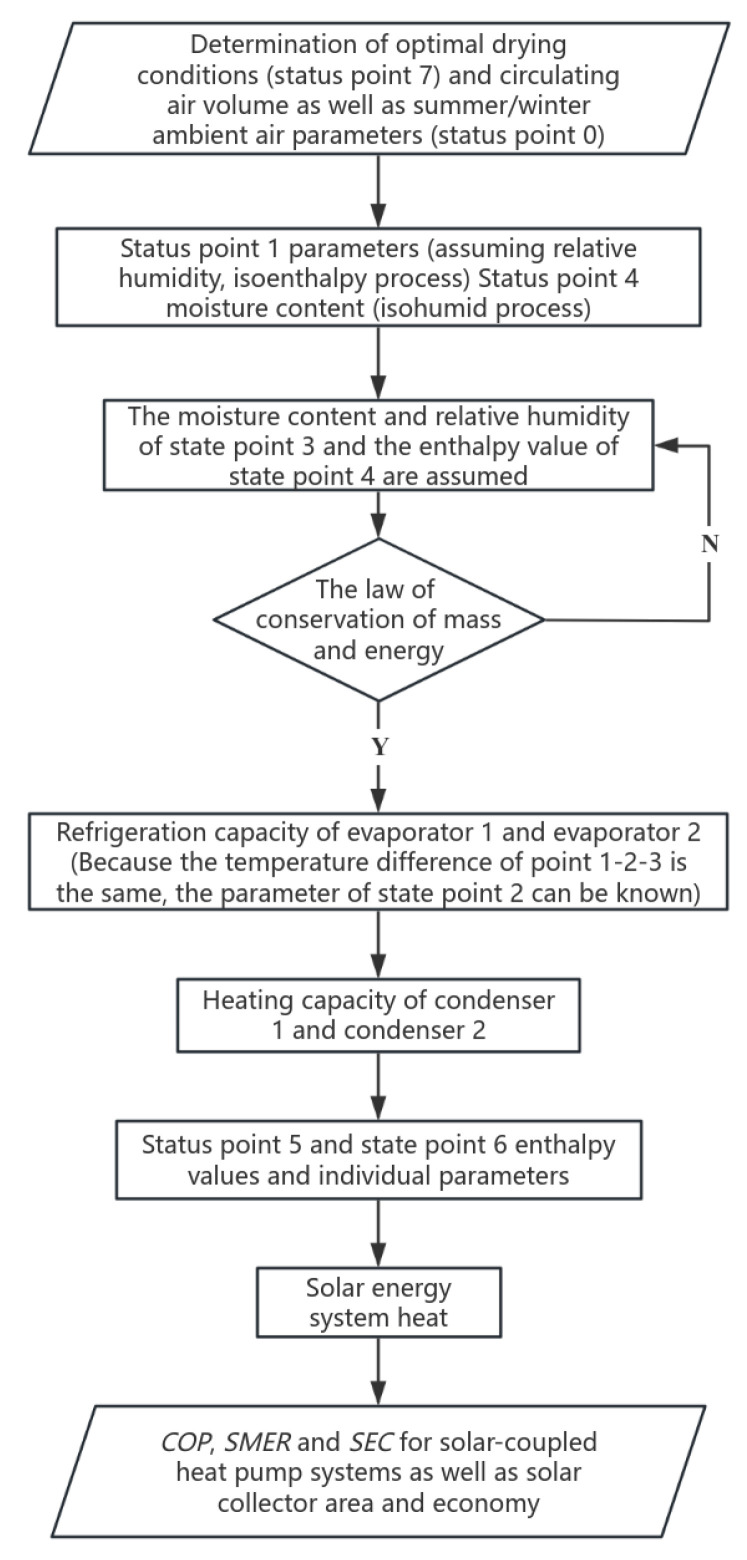
System performance calculation flowchart.

The total heat required in the drying process is composed of three parts, including the heat of the heating material, the latent heat of water evaporation, and the heat of heating the supply air [24]. In the tomato drying process, the heat loss is estimated to be 15%. The total heat of the system includes the heat produced by the heat pump and the heat collected by the solar collectors. The calculation process is shown in Figure 3.

The computational logic flowchart serves as a visual representation of the energy flow and key processes involved in the drying system. In this context, it outlines the calculation steps that determine the total heat required for the tomato drying process, accounting for heat losses, energy inputs, and the interactions between the heat pump and solar collectors.

## 3. Results and Discussion

### 3.1. Analysis of Drying Characteristics

**Figure 4:**
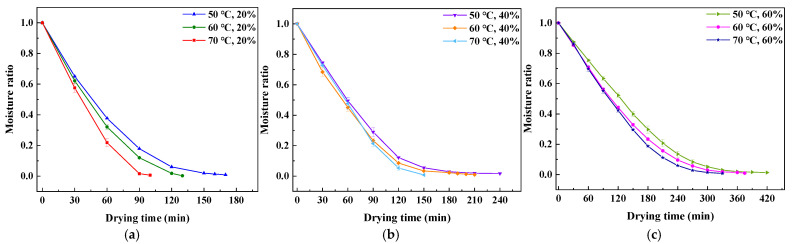
Moisture ratio curve: (**a**) *RH* = 20%; (**b**) *RH* = 40%; and (**c**) *RH* = 60%.

Figure 4 illustrates the hot air drying ratio of tomato slices (*p* < 0.05). The result shows a decreasing trend in the moisture ratio over the drying time, the drying time required to reach the target moisture content decreases as the drying temperature increases and the *RH* decreases. The dynamic moisture ratio changes observed during the hot air drying of tomato slices are similar to those reported for conventional solids such as potato slices [24] and apple slices [25].

**Figure 5:**
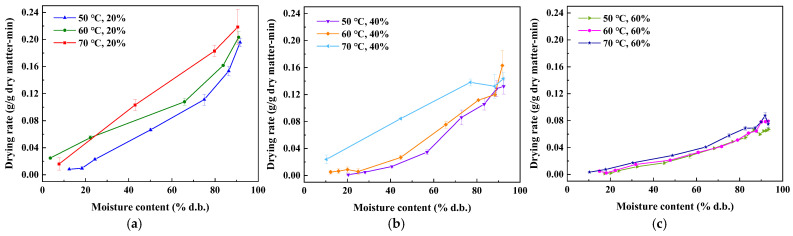
Drying rate curve: (**a**) *RH* = 20%; (**b**) *RH* = 40%; and (**c**) *RH* = 60%.

Figure 5 shows the *DR* versus *MC* of tomato slices dried in hot air. It was found that for the same moisture content, higher drying temperatures will accelerate the *DR* when the *RH* of the hot air is the same. Conversely, for the same temperature conditions, higher *RH* will reduce the *DR*.

Higher temperatures accelerate water evaporation, while lower *RH* creates a greater moisture gradient, increasing the air’s capacity to absorb moisture. Under these conditions, water molecules diffuse more quickly from the surface of the material into the air, thereby speeding up the overall drying process. In addition, at the beginning of the drying, the higher *DR* is due to a faster mass transfer rate and evaporation of a large amount of free moisture on the tomato surface. For the rate of moisture, diffusion decreased at the final drying stage as there was a significant reduction in available moisture within the tomato slices [26].

### 3.2. Analysis of Drying Temperature

**Figure 6:**
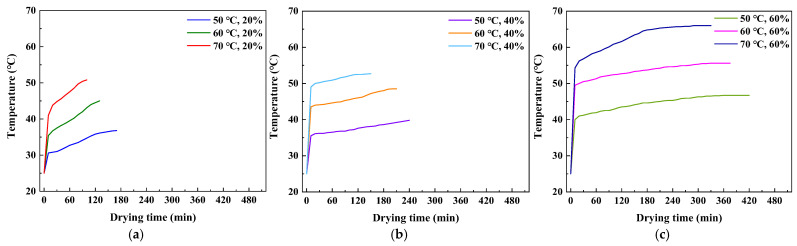
Temperature curve: (**a**) *RH* = 20%; (**b**) *RH* = 40%; and (**c**) *RH* = 60%.

Figure 6 illustrates the temperature changes in tomato slices during hot air drying. The process can be divided into two phases. The first phase is characterized by a rapid increase in temperature, while the second phase is characterized by a slower increase in temperature. In addition, at the same hot air temperature, the maximum temperature reached by the slices increases as the *RH* increases. This phenomenon can be analyzed on the basis of the drying characteristics of tomatoes. During the first phase, within the first five minutes, the temperature of the tomato slices increases most rapidly. In the initial drying stage, the low surface temperature of the slices results in significant heat absorption by the surface and boundary areas due to the temperature difference. This absorbed heat is used to heat the discs and evaporate surface moisture, resulting in a rapid rise in temperature. In the second stage, as the temperature of the tomato slices increases to a certain level, the rate of temperature increase gradually slows down. As the drying process progresses, the temperature difference between the heat source and the tomato slices gradually decreases, and the residual moisture content in the tomato slices also decreases, resulting in reduced heat transfer efficiency and a slower rate of temperature increase [27].

In addition, at the same temperature, a higher *RH* will result in a higher final temperature of the tomatoes. This is because higher *RH* increases the enthalpy of the hot air and the convective heat transfer coefficient. According to Fourier’s law of heat conduction, the rate of surface temperature increase in tomatoes is faster at higher *RH*. Once the surface reaches a higher temperature, the temperature gradient between the surface and the interior of the tomato increases, causing the internal temperature to rise rapidly and reach a higher level, closer to the drying hot air temperature.

### 3.3. Analysis of Effective Diffusion Coefficient of Moisture

From the data in Table 3, it can be seen that *D*_eff_ increases with increasing temperature and decreases with increasing RH. Firstly, the frequency of collisions between water molecules increases as the drying temperature increases. This results in more thermal energy being transferred to the water molecules in the tomato, which in turn increases the frequency of molecular transitions. As a result, water molecules can more easily overcome intermolecular attractions, resulting in increased thermal movement and a higher diffusion rate. Secondly, as *RH* increases, the vapor pressure difference between the tomato surface and the drying hot air decreases. This reduction in vapor pressure difference hinders the evaporation of water from the surface and interior of the tomato, suppressing the mass transfer process and thereby reducing the effective diffusion rate of water [28].

### 3.4. Analysis of Activation Energy

In Table 4, the *E*_a_ values at different *RH* conditions are listed. Research shows that the lower the *RH*, the higher the *E*_a_, indicating that it is more difficult to initiate drying under lower *RH* conditions. The lower the *RH*, the slower the rate of temperature rise in the material and the smaller the vapor pressure difference between the surface of the material and the environment. Therefore, more energy is required to overcome the barrier at the start of the drying process [29].

### 3.5. Analysis of Product Quality

#### 3.5.1. Color

**Figure 7:**
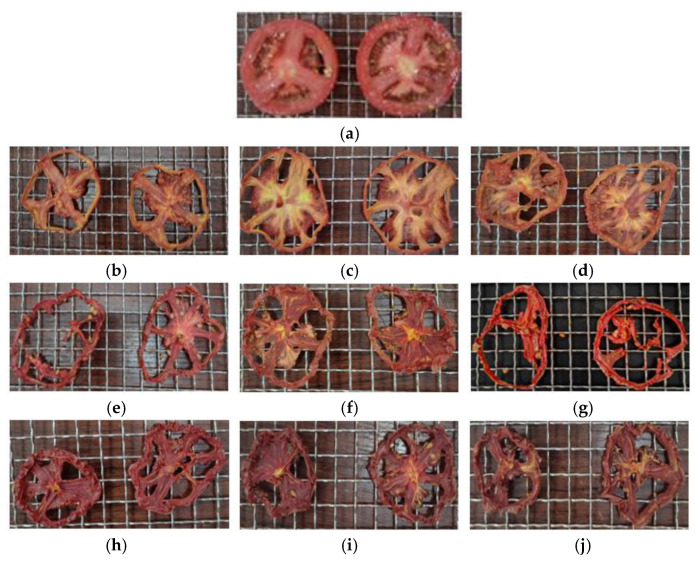
Color under different drying conditions: (**a**) initial color; (**b**) 50 °C, 20%; (**c**) 60 °C, 20%; (**d**) 70 °C, 20%; (**e**) 50 °C, 40%; (**f**) 60 °C, 40%; (**g**) 70 °C, 40%; (**h**) 50 °C, 60%; (**i**) 60 °C, 60%; and (**j**) 70 °C, 60%.

Color measurements were conducted at the Technical Institute of Physics and Chemistry, Chinese Academy of Sciences (Langfang, China). The observer used a colorimeter with a 10° viewing angle to perform five measurements on the tomato pulp under incandescent lighting, with the average value taken as the final result. The results of the color measurements of tomato slices dried under different drying conditions are shown in Table 5. The fresh tomato slices had color values of *L* = 33.5 ± 0.84, *a* = 16.62 ± 0.83, and *b* = 14.47 ± 0.65. The color of tomato slices under different drying conditions is shown in Figure 7 (*p* < 0.05).

Color is an important indicator of the quality of dried tomato products; the closer the color of the dried slices is to that of fresh tomatoes, the better the quality. The L*, a*, and b* values of dried tomato slices are related to the degree of browning. It can be observed that the tomato slices dried at 50 °C and 20% *RH* have the closest color similarity to fresh tomato slices, with the lowest ∆*E* value and the lowest *BI*. The reasons for this, based on the composition of the tomatoes, are as follows: Firstly, sugars and amino acids. Higher hot air temperature and *RH* lead to an increase in the temperature of tomato slices, which in turn accelerates the Maillard reaction between sugars and amino acids, forming brown compounds that affect color. If the drying temperature is too high, the color change is intensified, resulting in a darker appearance of the tomato slices. Secondly, polyphenols and flavonoids. Under high temperature and high *RH* conditions, tomato slices can reach higher temperatures, which decreases the content of active compounds such as phenols and flavonoids. These compounds have antioxidant properties and help preserve the natural color of the material [30].

#### 3.5.2. Flavonoid and Phenol

**Figure 8:**
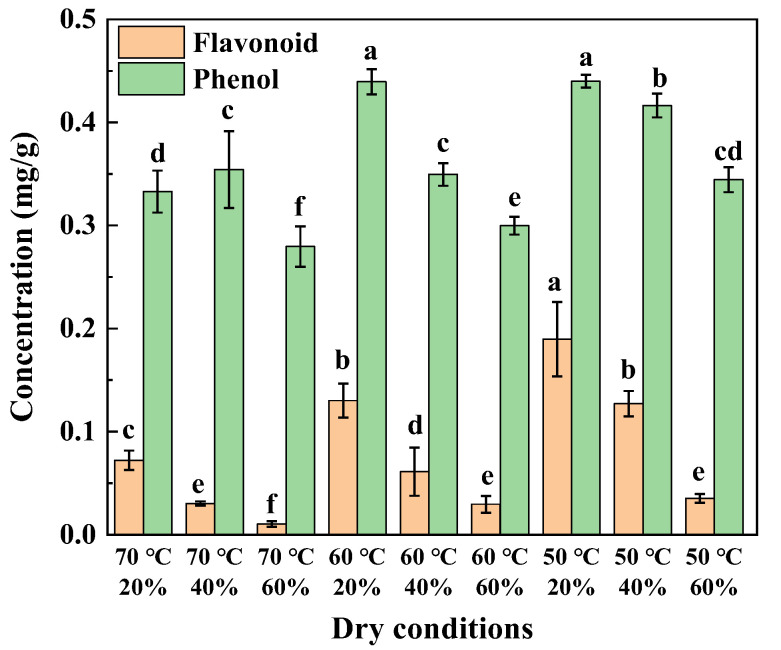
Content of flavonoids and phenols (Letters are salient analysis).

As shown in Figure 8 (*p* < 0.05), when the relative humidity (*RH*) of the hot air is constant, the total phenolic content in tomato slices decreases as the hot air temperature increases, which is consistent with studies on ginger, leafy vegetables, and red rice. When the hot air temperature is constant, higher *RH* leads to a lower total phenolic content, which is consistent with the results of onion studies. Under the conditions of 50 °C hot air temperature and 20% *RH*, the tomato slices had the highest total phenolic content of 0.4399 mg/g. The increase in total phenolic content is associated with increased degradation of phenolic compounds bound in the cell wall. As the hot air temperature and *RH* increase, the temperature of the tomato slices also increases. The high-temperature treatment disrupts the ester bonds between phenolic compounds and the cell wall, leading to the degradation of phenolic substances. The higher the temperature of the tomato slices, the greater the disruption of these ester bonds, resulting in a decrease in phenolic compounds [31].

If the *RH* of the hot air remains constant, the flavonoid content shows a decreasing trend as the temperature increases from 50 °C to 70 °C. Similarly, at a constant hot air temperature, an increase in *RH* leads to a decrease in flavonoid content. These results are consistent with those obtained in studies on tomato [32]. Under the combined conditions of 50 °C hot air temperature and 20% *RH*, the total flavonoid content in tomato slices reached a maximum of 0.1897 mg/g. This phenomenon is attributed to the elevated *RH* of the hot air, which increases the temperature of the tomato slices, promotes cell wall rupture, and accelerates the degradation of flavonoid compounds, thereby reducing the total flavonoid content.

### 3.6. Visualize the Heatmap

**Figure 9:**
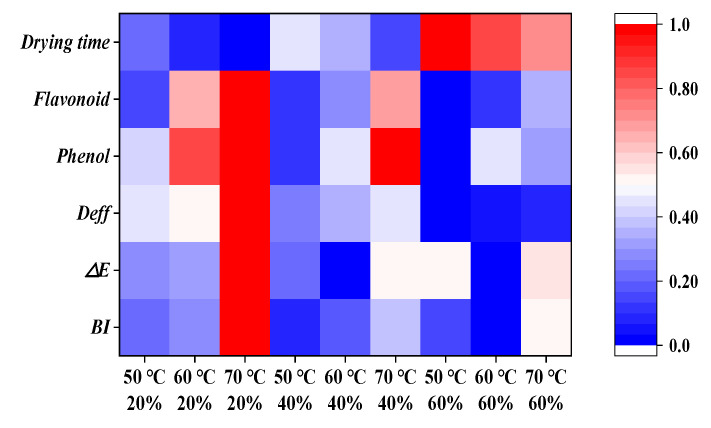
Visualization heat map of evaluation indicators.

Figure 9 presents a cluster analysis of various evaluation indices for tomatoes under different drying conditions, with red and blue representing the magnitude of the values. Drying time, activation energy (*E*_a_), and effective diffusivity (*D*_eff_) collectively influence the efficiency and outcome of the drying process. Conditions with high *D*_eff_ and low *E*_a_ result in shorter drying times. Indicators such as total flavonoid content, total phenolic content, browning index, and color difference are closely linked to product quality and are significantly influenced by temperature and humidity. Optimizing drying conditions helps preserve flavonoids and phenolic compounds while minimizing browning and color changes. By adjusting temperature and humidity, shorter drying times, higher moisture diffusivity, lower *E*_a_, and better retention of flavonoids and phenolic content can be achieved, reducing browning and color differences, ultimately enhancing product quality. The optimal drying process has been identified at 70 °C and 20% relative humidity.

### 3.7. Analysis of System Performance

#### 3.7.1. Power

**Figure 10:**
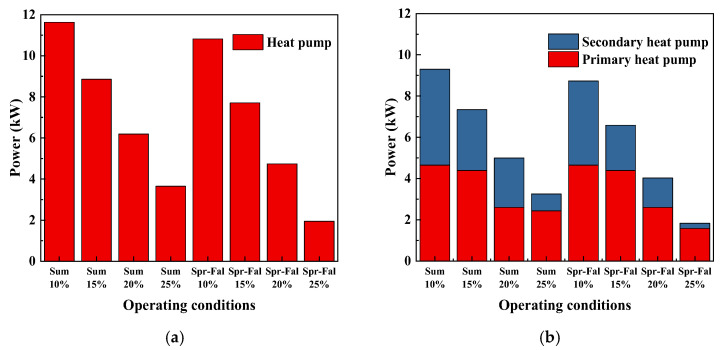
Comparison of heat pump system power in different seasons and fresh air volumes: (**a**) single-stage SAHPD; (**b**) double-stage SAHPD.

Figure 10 compares the power of the multi-stage heat pump system in different seasons with different fresh air flows, The power of the double-stage heat pump is lower than that of the single-stage heat pump. Due to the higher ambient temperature in summer compared to spring and autumn, the air contains more moisture and has a higher enthalpy. According to the principle of conservation of mass, the air at the evaporator outlet has a lower moisture content and lower enthalpy, resulting in a higher cooling capacity and higher power consumption of the heat pump. At the same time, the air entering the condenser has a lower temperature and enthalpy. Based on the principle of energy conservation, the enthalpy of the mixed air is higher after mixing with fresh air, which reduces the amount of heat provided by the solar energy system. If the total heat demand of the system remains constant, a smaller volume of fresh air means that more waste heat is recovered, increasing the heat output of the heat pump and reducing the heat supplied by the solar energy system. The heat pump output increases [33].

#### 3.7.2. *COP*

**Figure 11:**
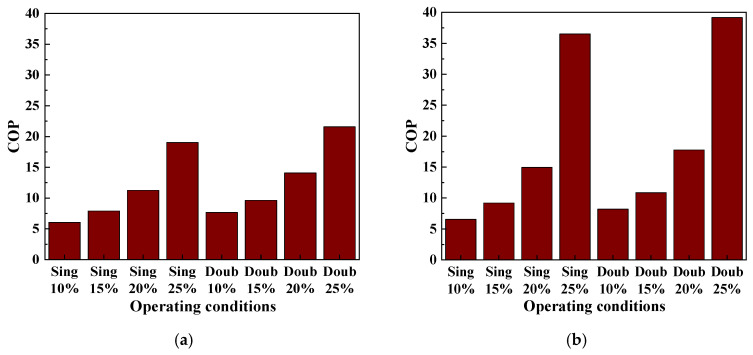
Comparison of *COP* in different seasons and fresh air volumes: (**a**) in summer; (**b**) in spring and autumn.

Figure 11 compares the *COP* of the multi-stage heat pump system under the same conditions. It can be seen that the *COP* of the double-stage SAHPD system is significantly higher than the single-stage system in all seasons, especially when the fresh air flow rate is high. The maximum *COP* is 21.58 in summer and 39.16 in spring/autumn. As the heat pump output is lower in spring and autumn than in summer, the *COP* is also higher. The effect of different fresh air volumes on the *COP* is also significant. The *COP* of the system increases with the amount of fresh air and peaks at 25% fresh air.

#### 3.7.3. Carbon Emissions

**Figure 12:**
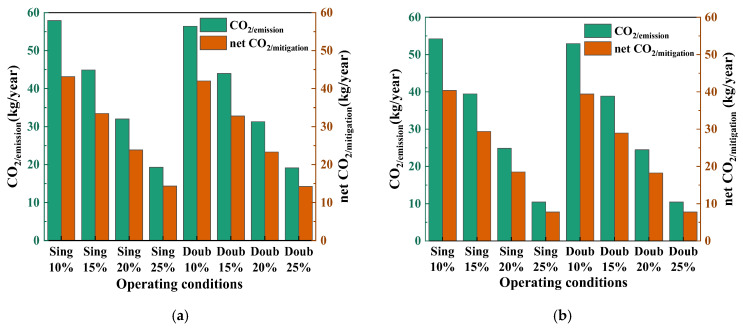
Comparison of *CO*_2/emission_ and net *CO*_2/mitigation_ in different seasons and fresh air volumes: (**a**) in summer; (**b**) in spring and autumn.

*CO*_2/emission_ and net *CO*_2/mitigation_ saving, shown in Figure 12, is very attractive to investors and government policy. The analysis shows that in summer and spring/autumn, the *CO*_2/mitigation_ of the double-stage SAHPD system is lower than that of the single-stage SAHPD system. The carbon emissions of the system are lower in spring and autumn than in summer. In addition, in spring and autumn, the *CO*_2/mitigation_ is the lowest when the fresh air volume is 25%. In summer, the *CO*_2/emission_ of the double-stage SAHPD at 25% fresh air is 19.11 kg/year. The net *CO*_2/mitigation_ reduction is 14.24 kg/year. In spring and autumn, the *CO*_2/emission_ of the system with 25% fresh air is 10.45 kg/year. The net *CO*_2/mitigation_ reduction is 7.78 kg/year.

#### 3.7.4. *SMER* and *SEC*

**Figure 13:**
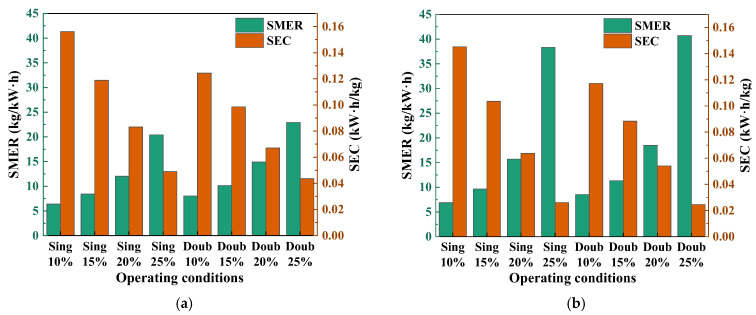
Comparison of *SMER* and *SEC* in different seasons and fresh air volumes: (**a**) in summer; (**b**) in spring and autumn.

Figure 13 shows the performance comparison of the system at different fresh air volumes and seasons. In the semi-open SAHPD system, the *SMER* increases as the fresh air volume increases, while the *SEC* decreases. The *SMER* is significantly higher in spring and autumn than in summer, while the *SEC* is significantly lower than in summer. For the double-stage SAHPD system, the *SMER* reaches a maximum of 22.92 kg/kWh and the *SEC* reaches a minimum of 0.043 kWh/kg at a fresh air rate of 25% in summer conditions. In spring and autumn conditions, the *SMER* peaks at 40.7 kg/kWh and the *SEC* drops to 0.024 kWh/kg.

This is because the increase in fresh air volume and the decrease in temperature in spring and autumn reduce the performance of the heat pump system, while the total dehumidification capacity remains unchanged. According to the calculation formulas for *SMER* and *SEC*, as the capacity decreases, the *SMER* gradually increases and the *SEC* gradually decreases [34].

#### 3.7.5. Solar Collector and Expansion Tank

**Figure 14:**
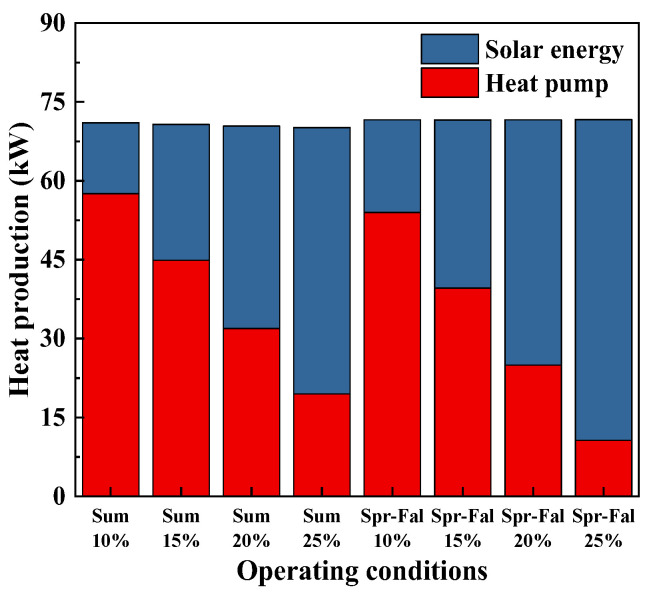
The heating capacity.

Based on the heating capacity of the solar-coupled dual-stage heat pump drying system under different fresh air flow conditions in spring, autumn, and summer (as shown in Figure 14), and assuming a solar radiation intensity of 8000 kJ/m^2^ with a water temperature increase of 30 °C, the effective area of the solar collector and the total water volume of the storage tank are calculated according to the formulas in Section 2.4.2 and Section 2.4.3. The results are given in Table 6.

## 4. Conclusions

This study analyzes the drying characteristics and quality of *Solanum lycopersicum* L. under nine different hot-air drying conditions and evaluates the thermodynamic and economic performance of a novel semi-open double-stage solar-assisted heat pump drying (SAHPD) system based on the optimized drying process. The results show that higher relative humidity (*RH*) at the same drying temperatures decrease the effective moisture diffusivity, extending drying times and lowering activation energy. However, higher *RH* helps preserve the natural color of fresh tomatoes and maintains higher material temperatures, which aids in retaining phenolics and flavonoids. The optimal drying conditions for tomato slices were found to be 70 °C and 20% *RH*, resulting in a drying time of 100 min.

The double-stage SAHPD system outperforms the single-stage system by offering more precise humidity control and better solar energy utilization. In summer, it achieved a 13% increase in *COP*, a 12% improvement in *SMER*, and a 10% reduction in *SEC*. In spring and autumn, these metrics improved by 7%, 6%, and 6%, respectively. Under typical spring/autumn conditions (25 °C, 55% *RH*) with 25% fresh air intake, the system reached a *COP* of 39.16 and a peak *SMER* of 40.7 kg/kWh, while limiting *CO*_2/emissions_ to 10.45 kg/year, resulting in a net mitigation of 7.78 kg/year. In summer (30 °C, 60% *RH*), the system achieved a maximum *COP* of 21.58 and *SMER* of 22.92 kg/kWh, reducing *CO*_2/emissions_ by 8.66 kg/year, with a net mitigation of 6.46 kg/year.

The findings highlight the effectiveness of the double-stage SAHPD system in improving energy efficiency, product quality, and reducing carbon emissions. The system’s ability to optimize drying performance across different environmental conditions demonstrates its potential for widespread industrial applications, especially in energy-efficient food drying. Future research should focus on system scalability, integration with alternative renewable energy sources, and the development of real-time adaptive control strategies to further enhance its sustainability and economic viability.

## Figures and Tables

**Table 1:** Specific operational steps.

Reagent Name	Control Tube	Measurement Tube	Measurement Tube	Measurement Tube
Sample to be measured (μL)	50	50	-	-
Standard solution (μL)	-	-	50	-
Distilled water (μL)	-	-	-	50
Reagent 1 (μL)	-	250	250	250
Vortex and let sit at room temperature for 2 min				
Reagent 2 (μL)	250	250	250	250
Distilled water (μL)	700	450	450	450

**Table 2:** Specific operational steps.

Reagent Name	Control Tube	Measurement Tube	Measurement Tube	Measurement Tube
Sample to be measured (μL)	0.2	0.2	-	-
Standard solution (μL)	-	-	0.2	-
Distilled water (μL)	-	-	-	0.2
Reagent 1 (μL)	0.05	0.05	0.05	0.05
Vortex and let sit at room temperature for 5 min				
Reagent 2 (μL)	-	0.05	0.05	0.05
Vortex and let sit at room temperature for 5 min				
Reagent 3 (μL)	0.4	0.4	0.4	0.4
60% ethanol (mL)	0.35	0.3	0.3	0.3

**Table 3:** Effective water diffusion coefficient.

Temperature/°C	*RH*/%	Linear Regression Fits the Equation	*R* ^2^	*D*_eff_/(m^2^/s)
50	20	ln*MR* = −0.209 − 0.00040*t*	0.9741	4.05 × 10^−9^
60		ln*MR* = −0.209 − 0.00043*t*	0.9224	4.35 × 10^−9^
70		ln*MR* = −0.209 − 0.00069*t*	0.9215	7.00 × 10^−9^
50	40	ln*MR* = −0.209 − 0.00028*t*	0.9824	2.83 × 10^−9^
60		ln*MR* = −0.209 − 0.00033*t*	0.9871	3.40 × 10^−9^
70		ln*MR* = −0.209 − 0.00029*t*	0.9120	4.06 × 10^−9^
50	60	ln*MR* = −0.209 − 0.00015*t*	0.9637	1.52 × 10^−9^
60		ln*MR* = −0.209 − 0.00017*t*	0.9638	1.74 × 10^−9^
70		ln*MR* = −0.209 − 0.00019*t*	0.9427	1.96 × 10^−9^

**Table 4:** Activation energy.

Temperature/°C	*RH*/%	*R* ^2^	*E*_a_/(kJ/mol)
50	20	0.9855	25.22
60			
70			
50	40	0.9996	16.53
60			
70			
50	60	0.9999	11.70
60			
70			

**Table 5:** Color evaluation index (Superscript is significance analysis).

Temperature/°C	*RH*/%	$L^{*}$	$a^{*}$	$b^{*}$	ΔE	BI
50	20	33.89 ± 0.13 ^a^	21.06 ± 1.01 ^b^	19.55 ± 0.61 ^ab^	7.03 ± 1.14 ^c^	124
60		25.55 ± 0.57 ^cd^	13.85 ± 1.50 ^d^	18.31 ± 0.70 ^b^	9.17 ± 0.18 ^bc^	150
70		27.95 ± 1.04 ^bc^	22.42 ± 0.73 ^ab^	17.36 ± 1.11 ^b^	8.83 ± 0.49 ^bc^	144
50	40	29.47 ± 0.84 ^b^	23.12 ± 0.80 ^ab^	16.81 ± 1.16 ^b^	8.35 ± 0.56 ^bc^	132
60		27.31 ± 1.14 ^bc^	17.91 ± 0.63 ^c^	17.73 ± 1.23 ^b^	7.23 ± 1.46 ^bc^	141
70		27.04 ± 0.94 ^bc^	23.23 ± 0.60 ^ab^	18.34 ± 0.29 ^b^	10.31 ± 0.34 ^c^	159
50	60	29.97 ± 1.37 ^b^	25.52 ± 0.27 ^a^	17.43 ± 0.23 ^b^	10.40 ± 0.29 ^b^	138
60		28.99 ± 0.74 ^b^	22.56 ± 1.03 ^ab^	21.72 ± 0.86 ^a^	10.65 ± 1.51 ^b^	172
70		22.65 ± 0.99 ^d^	22.10 ± 0.94 ^b^	20.00 ± 1.35 ^ab^	13.55 ± 0.16 ^a^	219

**Table 6:** Calculation of collector efficiency and total water volume in summer under different fresh air volumes.

	Fresh Air Volumes	10%	15%	20%	25%
Summer	Solar collector heat supply (kJ)	13.48	25.8	38.47	50.63
	Solar radiation intensity (kJ/m^2^)	8000.00	8000.00	8000.00	8000.00
	Effective area of solar collector (m^2^)	17.24	32.99	49.19	64.74
	Water temperature increase (°C)	30.00	30.00	30.00	30.00
	Water flow rate (m^3^/h)	0.39	0.74	1.11	1.46
	Total water volume (m^3^)	0.65	1.24	1.85	2.44
Spring/autumn	Solar collector heat supply (kJ)	17.6	31.94	46.62	61
	Solar radiation intensity (kJ/m^2^)	8000.00	8000.00	8000.00	8000.00
	Effective area of solar collector (m^2^)	22.51	40.84	59.62	78.01
	Water temperature increase (°C)	30.00	30.00	30.00	30.00
	Water flow rate (m^3^/h)	0.51	0.92	1.35	1.76
	Total water volume (m^3^)	0.85	1.54	2.25	2.94

## Data Availability

The original contributions presented in the study are included in the article, further inquiries can be directed to the corresponding authors.
